# Using social media for health education and promotion: a pilot of WeChat-based prize quizzes on China national malaria day

**DOI:** 10.1186/s12936-022-04404-2

**Published:** 2022-12-13

**Authors:** Yi Wang, Chengyuan Li, Jiayao Zhang, Mengmeng Yang, Guoding Zhu, Yaobao Liu, Jun Cao

**Affiliations:** 1 National Health Commission Key Laboratory of Parasitic Disease Control and Prevention, Jiangsu Provincial Key Laboratory On Parasite and Vector Control Technology, Jiangsu Institute of Parasitic Diseases, Wuxi, 214064 Jiangsu People’s Republic of China; 2 Center for Global Health, School of Public Health, Nanjing Medical University, Nanjing, China

**Keywords:** Malaria, Social media, WeChat, Sojump, Malaria day, Health education

## Abstract

**Background:**

Imported malaria cases remains a key health concern, especially during the COVID-19 pandemic. Providing accurate health information is important to improving people’s awareness of malaria. WeChat is an excellent social media tool for health information dissemination, especially during the pandemic. This study explored the effect of malaria knowledge dissemination via a WeChat public account.

**Methods:**

A questionnaire for data collection was constructed using the online survey tool Sojump. Questionnaires were sent to users who followed the Jiangsu institute of Parasitic Disease WeChat public account during the National Malaria Day 2021 period. A small incentive (WeChat Red Packet) was distributed to everyone who answered the questionnaire correctly on time.

**Results:**

A total of 13,169 valid questionnaires were collected during the China National Malaria Day period. Questions in which participants focused mainly on information pertaining to themselves, such as infection, symptoms, and epidemic areas, reached highest accuracy (above 90%). Questionnaires were submitted through smartphones and most of them were completed during the period of 4 days from April 23 to April 26, 2021 when a WeChat Red Packet was offered. The accuracy of responses was related to bolded words and location and number of knowledge points that were shown at the beginning of the questionnaire. The number of users of the WeChat public account in question increased from 5961 to 12,339 in just 4 days of the activity.

**Conclusion:**

A WeChat public account is a convenient and accessible tool for spreading malaria-related health information to the public. Distribution of incentives (Red Packets) can effectively increase public attention to popular science and health information and activities.

**Supplementary Information:**

The online version contains supplementary material available at 10.1186/s12936-022-04404-2.

## Background

Malaria elimination has made remarkable progress in China and the country was certified as malaria free by the World Health Organization (WHO) in 2021. However, with increasing numbers of Chinese migrant workers and more business, tourism, and international exchanges in recent years, the number of imported malaria cases from other countries remains high [1]. A low degree of public knowledge about malaria has been observed in many studies conducted in China, with particularly weak knowledge on its prevention. Young people and residents living far from township hospitals tend to have poorer knowledge of malaria [2]. Malaria awareness among travellers from China remains lower than the national goal [3]. Students at elementary and high schools also have poor awareness of malaria [4]. Health education has long-term effects on disease control and is an indispensable component of malaria elimination [5].

The benefits of health education in the prevention of malaria had been demonstrated in Thailand [6]. The experience of schistosomiasis control in China has also shown that the implementation of a health education programme can improve people’s knowledge level and change their attitudes [7]. Traditional media (e.g. bulletin boards, newspapers, shopping bags, leaflets) have played an important role in influencing public awareness of malaria; however, one of the challenges is difficulty to achieve the expected effect [8]. Therefore, use of a variety of media and means to improve the effect of health education is necessary to meet the needs of various target populations.

Social media is becoming an important platform for information dissemination. This is believed to have direct implications for health education, prompting new opportunities to use social media to impact disease prevention. Social media attracts the largest proportion of Internet users and this is likely to continue to grow, making them an obvious target for maximizing the reach and impact of health education [9]. Social media may have the capacity to reach a wider audience than traditional media. A broad range of people including the general public, patients, and health professionals can use social media to communicate about health issues [10]. According to the 47th China Statistical Report on Internet Development (CNNIC), the number of Chinese “netizens” (citizens who use the Internet) has reached 989 million, mobile phone Internet users has reached 986 million, and the percentage of Internet users who surf the Internet via mobile phone is as high as 99.7%. Netizens aged 20–50 years are the main population age group, accounting got 57.1% of all users. Among the netizens in China, 59.6% have junior high school education or below and only 19.8% have a college degree or above [11]. This provides an opportunity to conduct health education through social media in China.

WeChat is a free mobile device application that was released in 2011 and has become one of the most popular social media platforms in China. It presents complex information by instant text, voice message, video, and graphics, providing an accessible way to spread health information to the public. WeChat public accounts, one of functions of WeChat, can be used by government, companies, and organizations to provide information. Health information through WeChat public accounts provided by professional institutions plays an important role in improving public health literacy. For example, it has been applied in health education for heart disease [12], cancer [13], and diabetes [14].

Low public participation is one of the biggest challenges for health education programs [15]. WeChat has created a featured incentive named “WeChat Red Packet” and through which people can receive a minor bonus (up to 2RMB). National Malaria Day on April 26, 2021 had the theme “Preventing re-establishment and consolidating the achievements of malaria elimination”.

This study explored the effect of malaria knowledge dissemination via a WeChat public account and whether the use of WeChat Red Packet as an incentive increased engagement with malaria health information during the National Malaria Day period in China.

## Methods

### WeChat public account

**Fig. 1:**
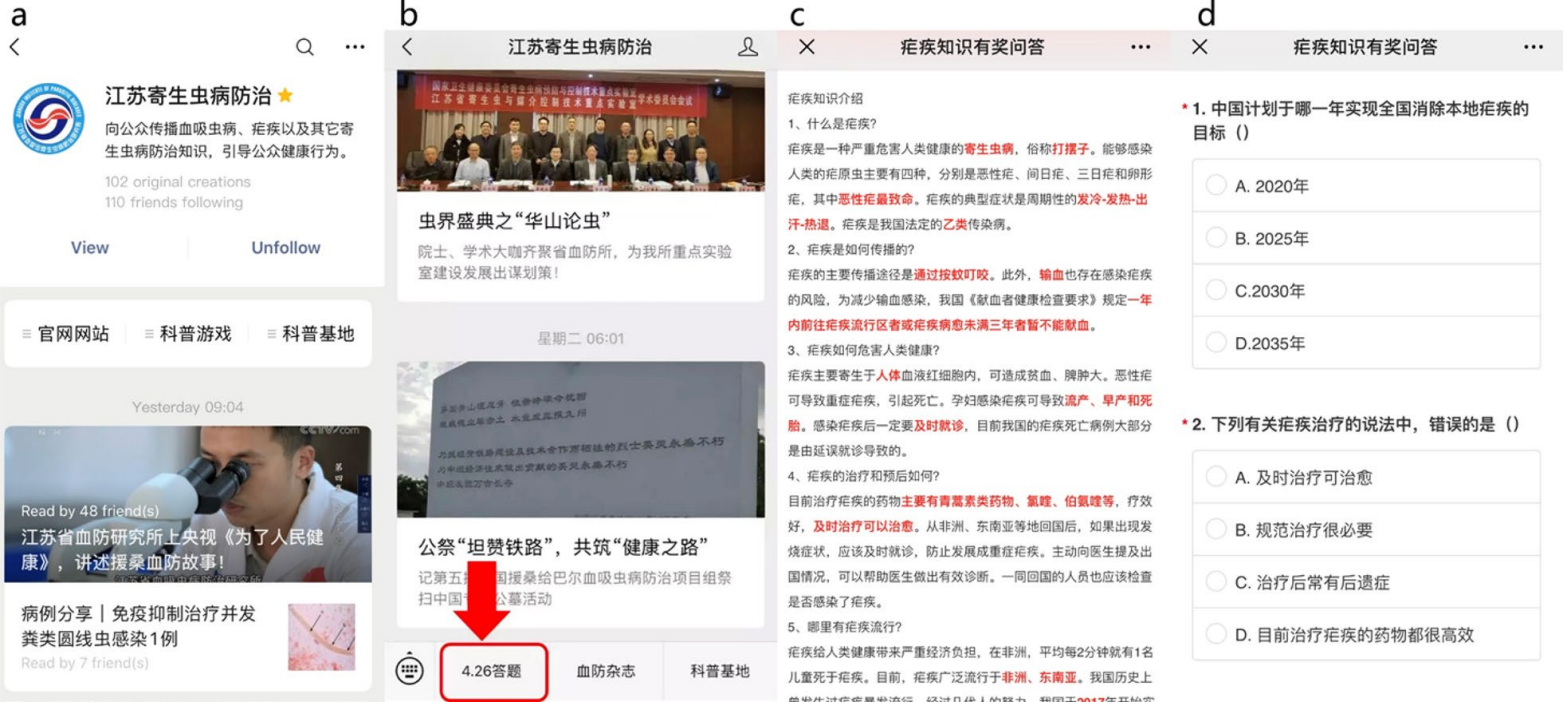
The activity interface. Note: **a** frontpage of the WeChat public account; **b** entry to the survey; **c** basic malaria knowledge points; **d** questionnaire interface

The WeChat public account (Parasitic Disease Control and Prevention in Jiangsu) used in the activity is the official account of Jiangsu Institute of Parasite Disease (JIPD). The account was established in 2016 and health information on parasitic diseases is regularly broadcast on the account by JIPD. Followers of the WeChat public account receive health information related to parasitic diseases (Fig. 1).

### Activity design and implementation

A questionnaire for data collection was constructed using a free online survey tool named Sojump (http://www.sojump.com). The questionnaire was titled “Questionnaire on knowledge of malaria” and comprised 5 questions randomly selected from a bank of 24 questions (Additional File 1).

**Fig. 2:**
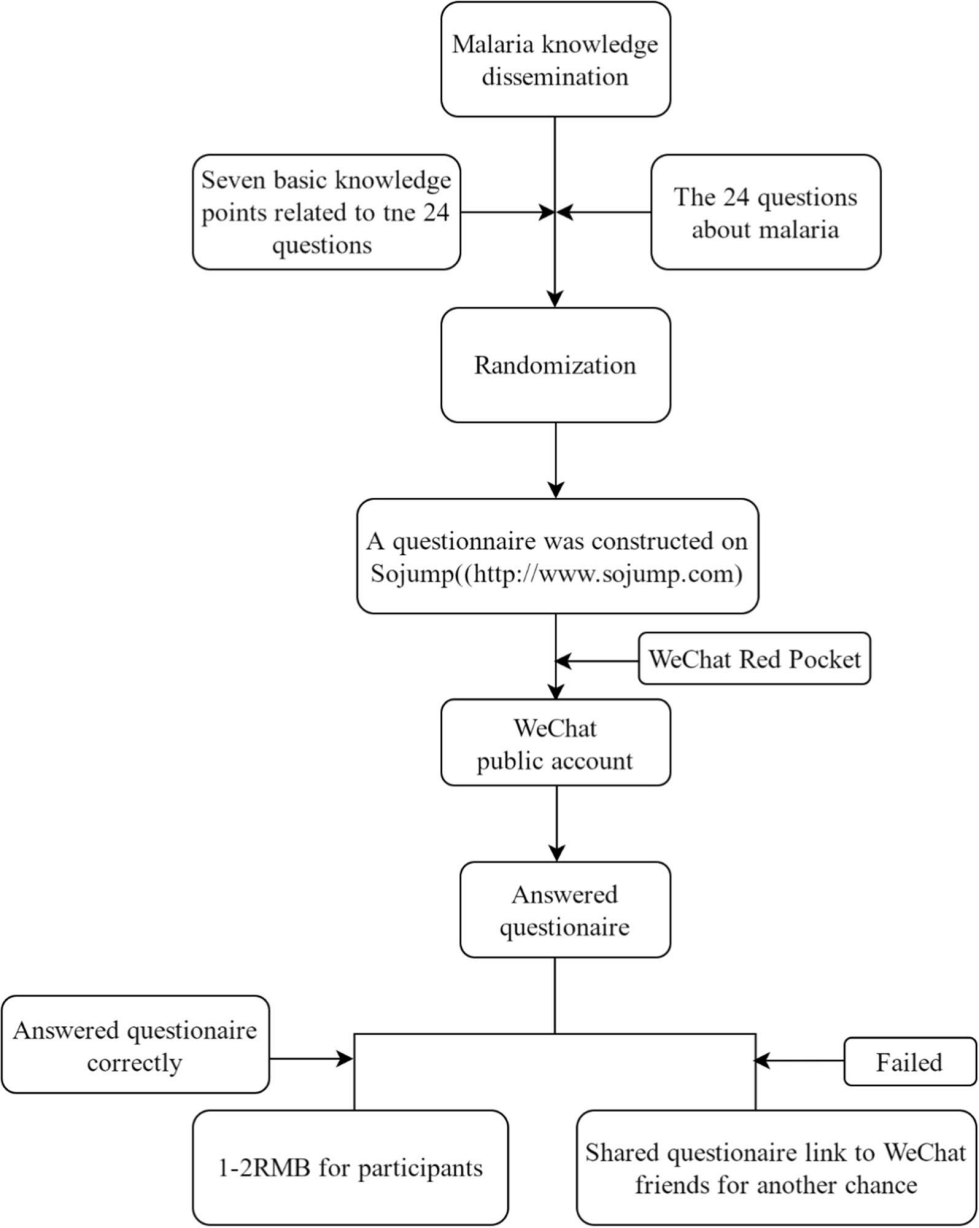
Flow diagram of the activity design

To increase public interest, incentives were provided by issuing a Red Packet. Participants who answered the questions correctly within 5 min received a bonus of 1–2 RMB. To expand the participation, each participant initially had only one opportunity to complete the activity but could share the questionnaire link to their WeChat friends to win another opportunity (Fig. 2).

Seven basic malaria knowledge points related to the question bank were presented at the beginning of the questionnaire for participants to learn (Additional File 2). The most important words of the knowledge points were highlighted in bold red characters to attract the attention of the participants. The questionnaires were sent to users who followed the WeChat public account. The activity started on April 23, 2021 and lasted until April 26, 2021.

### Data analysis

Data including graphical distribution, score, and response time were collected and analyzed when the questionnaires were submitted via Sojump. The data were analysed using Microsoft Excel 2016.

## Results

### General information

**Fig. 3:**
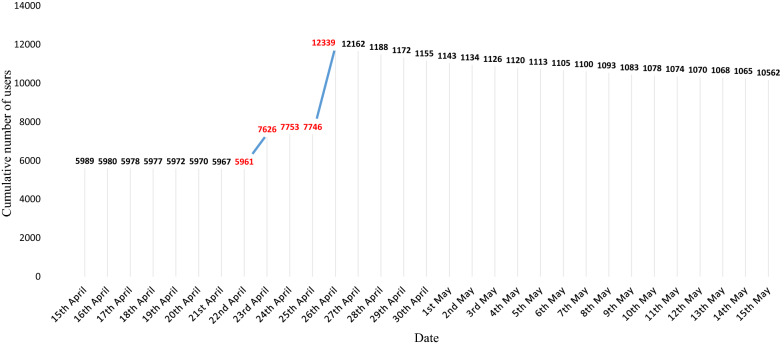
Change in the number of WeChat public account users

**Fig. 4:**
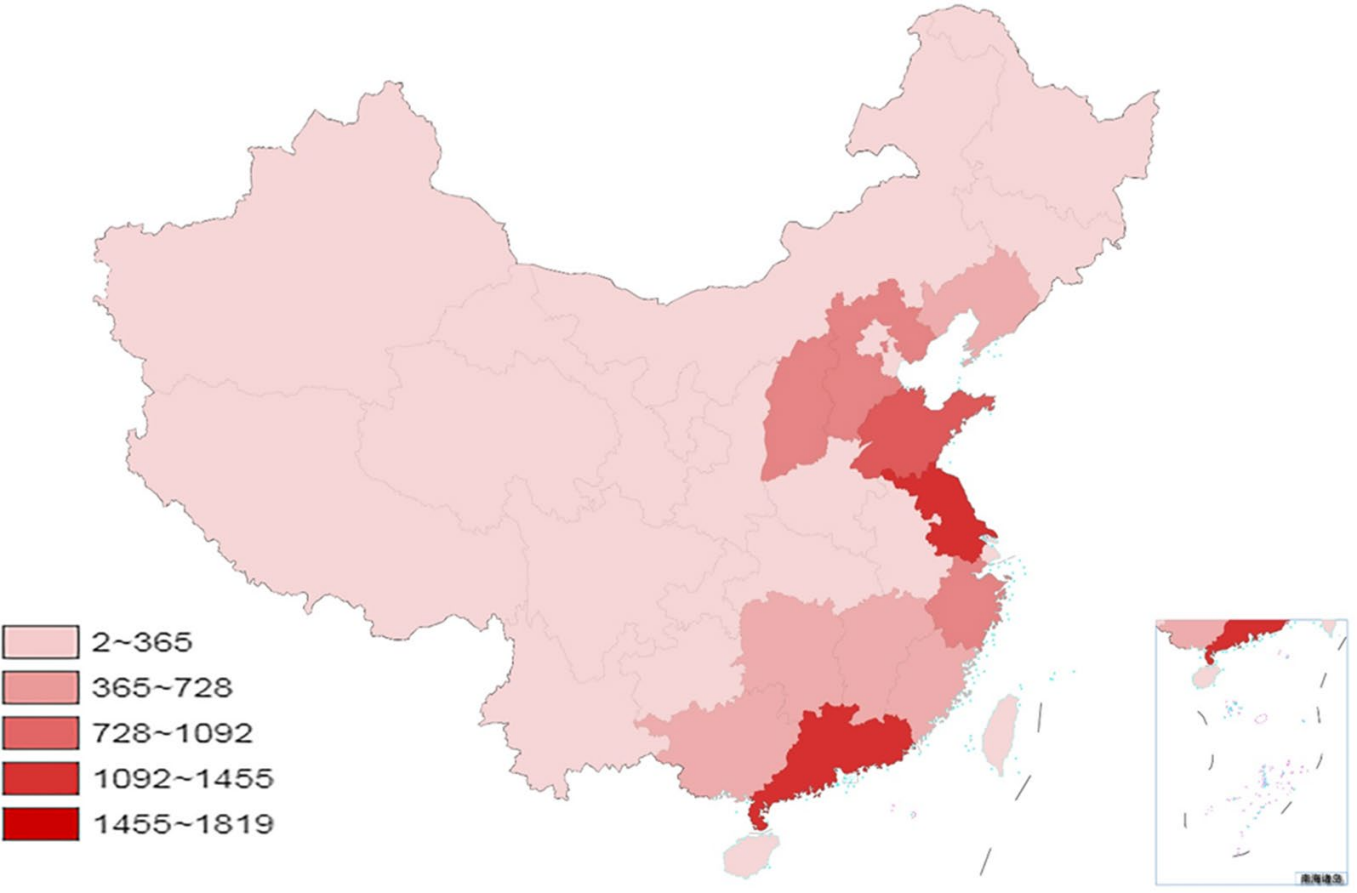
Geographical distribution of participants in China

A total of 13,169 valid questionnaires were collected from 32 provinces and autonomous regions across China. The number of WeChat public account users increased from 5961 to 12,339 in just 4 days of the activity (Fig. 3). The majority of participants were from Jiangsu, Guangdong, Shandong, Zhejiang, and Shanxi (Fig. 4). All questionnaires were submitted through smartphones.

**Fig. 5:**
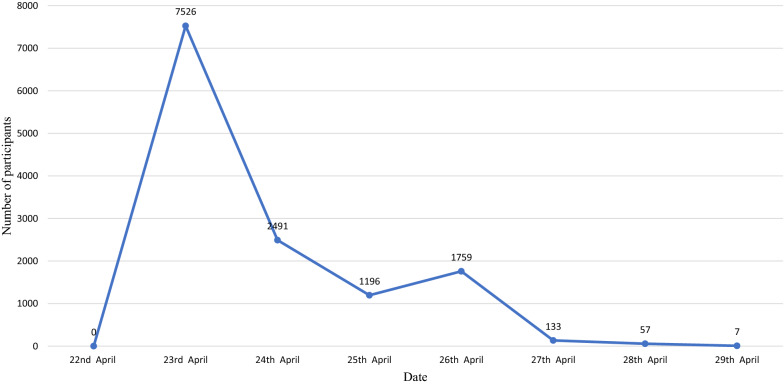
Daily number of participants from April 23 to April 26, 2021

The survey lasted 4 days and the bonus pool reached the largest on April 23. Most of questionnaires were completed between April 23 and 26, 2021 when the bonuses were offered. The number of participants decreased as the bonus share decreased (Fig. 5). A total of 6064 (46%) participants answered all five questions within the specified 5 min. The accuracy of the responses gradually increased over the 4 days.

### Correlation between accuracy and knowledge points

The accuracy of the answers depended mostly on the knowledge points we offered at the beginning of the questionnaire, specifically relating to bolded words and location and number of knowledge points. The information relating to the question with the lowest proportion of correct responses, “What is the isolation treatment for malaria patients?” (41.81%), was not presented in the knowledge points. The next lowest accuracy was for the question “What kind of mosquito spreads malaria?” which was answered correctly at only 50.55%; this reflected another knowledge point that was not highlighted. The accuracy of responses to the questions “When was Jiangsu Province formally assessed as having eliminated malaria?” and “When did China report no locally acquired malaria cases for an entire year?” was 62.45–70.13%, respectively. These two questions were presented at the fifth knowledge point, which had the most highlighted words. Questions were answered less accurately if some knowledge points were included at the same time. For example, the accuracy of “Which of the following statements about malaria treatment is false?” was 68.5%.

In addition, participants appeared to have better focus on information pertaining to themselves, such as on infection, symptoms, and epidemic areas, that was closely related to daily human life. For example, questions with higher than 90% accuracy in responses were those about the harm of malaria to pregnant women, what should be done if the individual is infected with malaria or has a fever, and the prevention of malaria (Table 1).

**Table 1:** Accuracy of question responses

Questions	Accuracy (%)	Degree
What harm does malaria cause to pregnant women?	95.11	Above 90%
What should I do if I have a fever after returning from Africa or Southeast Asia?	93.61	
What should I do if I am infected with malaria?	92.3	
How is malaria prevented?	90.74	
What kind of mosquito spreads malaria?	50.55	Below 60%
What is the isolation treatment for malaria patients?	41.81	

## Discussion

This study explored a new method for health education using quizzes with prizes on social media. A method was explored based on WeChat and the online survey tool Sojump for malaria information dissemination. A total of 13,169 valid questionnaires were collected from most of the regions of China, with the exception of two provinces and autonomous regions. Most of them were completed during the period in which a bonus was offered. Social media appeared to be convenient for information dissemination and the bonus was a critical incentive for the participants. The accuracy of responses to the questions was associated with the number of words presented as malaria knowledge points and the positions of the knowledge points presented. Participants were focused mainly on information pertaining to themselves and seemed not to read all the knowledge points carefully even if they were brief.

The method included four major strengths: (1) Administration of the questionnaire was via a WeChat public account, that of the JIPD, and could be done on particular dates. The dates was selected in anticipation of 2021 National Malaria Day to strengthen consciousness of malaria prevention. (2) The activity took a feedback loop design to ensure that participants have the correct information reinforced. Seven basic malaria knowledge points related to the question bank were presented before each questionnaire. The participants can learn the correct information again if they cannot answer question correctly for the first time. Because participants who only answered the questions correctly within 5 min can received a bonus. This differed from a traditional format, to maximize the efficiency of the dissemination of malaria knowledge. Most participants tended to read mostly the highlighted terms based on the results, even if the knowledge points were brief. (3) A 1–2RMB randomly distributed bonus was supplied to maximize participation. Less attention is given to health information delivered by WeChat when information is rich [16] and this provided an ideal opportunity to try a new approach. Potential access to a bonus played an important role in the study—the number of participants decreased significantly when the bonus was stopped. The largest number of participants in the activity was on the first day because the bonus was at its highest. Another method was trialed to attract more participants besides the bonus awarded directly for answering questions—participants could share the questionnaire link to their WeChat friends for another opportunity to answer questions if they did not win the bonus the first time. (4) The activity was constructed using an online survey tool and WeChat. They were both free and just a total of 3800 RMB bonus was paid. The average cost per questionnaire was only 0.28 RMB. It was cheaper than traditional media (e.g. bulletin boards, leaflets, brochures). Additionally as a follow-up of participants was not needed, this mode of delivery was suitable.

The number of Internet users in China’s rural areas presently stands at 309 million. Among the netizens in China, students are the large population. So, it is a suitable channel for reaching them with health communications. In China, people tend to obtain information passively from WeChat Moments, micro-blogs, and health-related web pages. WeChat Moments (personal updates and content), WeChat public accounts, and group chats are the main means through which users receive health information, because of their convenience and accessibility [17]. However, with the rapid development of society and social media, people have entered an era of overload of information that may not be useful or accurate [18]. Many people are attracted to clicking on articles specifically styled to attract attention and interest even though the content is not professional (“click-bait”). This means that individualized and professional health information provided by healthcare entities is even more crucial. Parasitic diseases are particularly neglected. Health education departments are well placed to make full use of WeChat public accounts to engage target users and increase the effectiveness of health information dissemination.

COVID-19 was declared a pandemic infection by the WHO on March 11, 2020 [19]. The pandemic has caused considerable impact on other diseases, including malaria. The process of malaria diagnosis had been lengthened because of clinical similarities between malaria and COVID-19 infection [20]; thus, it is even more important that people should understand about the existence of malaria, particularly when physician attention is diverted toward COVID-19. Social media has provided new channels for people to find and share information during the COVID-19 pandemic. WeChat official accounts are increasingly widely used by public health institutions for health information dissemination. Many studies on the use of WeChat for health information have focused on clinical medicine, nursing, and nutrition [21–23]. However, few studies have focused on parasitic disease, making this a novel study in the field.

This study also has some limitations. The study was Internet based, which meant the participants may have been of a higher socioeconomic background than the general public and thus not representative of the entire population. Other methods should be considered for those who do not have access to smartphones. In addition, it is not possible to accurately identify the target population. Other technological means can be used to disseminate health information to target populations, for example, e-commerce sites such as Amazon send key information targeted according to search history. Lastly, the number of WeChat account users decreased after the activity ended. High attention to malaria health education only lasted during the period April 23–26, 2021 when the bonus was available. This suggests that the activity should be repeated periodically.

## Conclusion

This study demonstrates the convenience and accessibility of the WeChat application for spreading health information. Distribution of Red Packets can effectively increase public attention to health and popular science information and activities. However, various media formats and modes of delivery should be considerate for different target populations. Media tools need to be explored further as a method of health education on imported malaria.

## Supplementary Information

**Additional file 1.** Question Bank.

**Additional file 2.** Malaria knowledge points.

## Data Availability

All relevant data are included in this report.

## Abbreviations

WHO: World Health Organization
JIPD: Jiangsu institute of parasitic diseases
NMEP: National malaria elimination programme
CNNIC: China Statistical Report on Internet Development

## References

[1] Zhou S, Li Z, Cotter C, Zheng C, Zhang Q, Li H (2016). Trends of imported malaria in China 2010–2014: analysis of surveillance data. Malar J.

[2] Tang S, Ji L, Hu T, Bishwajit G, Da F, Ming H (2016). Determinants of public malaria awareness during the national malaria elimination programme: a cross-sectional study in rural China. Malar J.

[3] Zhang M, Liu Z, He H, Luo L, Wang S, Bu H (2011). Knowledge, attitudes, and practices on malaria prevention among Chinese international travelers. J Travel Med.

[4] Yin J-h, Wang R-b, Xia Z-g, Zhou S-s, Zhou X-n, Zhang Q-f (2013). Students’ awareness of malaria at the beginning of national malaria elimination programme in China. Malar J.

[5] Lai S, Sun J, Ruktanonchai NW, Zhou S, Yu J, Routledge I (2019). Changing epidemiology and challenges of malaria in China towards elimination. Malar J.

[6] Okabayashi H, Thongthien P, Singhasvanon P, Waikagul J, Looareesuwan S, Jimba M (2006). Keys to success for a school-based malaria control program in primary schools in Thailand. Parasitol Int.

[7] Qian M-B, Zhou C-H, Zhu H-H, Zhu T-J, Huang J-L, Chen Y-D (2019). Assessment of health education products aimed at controlling and preventing helminthiases in China. Infect Dis Poverty.

[8] Aung PL, Pumpaibool T, Soe TN, Burgess J, Menezes LJ, Kyaw MP (2019). Health education through mass media announcements by loudspeakers about malaria care: prevention and practice among people living in a malaria endemic area of northern Myanmar. Malar J.

[9] Jahanbin K, Rahmanian F, Rahmanian V, Jahromi AS (2019). Application of Twitter and web news mining in infectious disease surveillance systems and prospects for public health. GMS Hyg Infect Control.

[10] Zhang X, Wen D, Liang J, Lei J (2017). How the public uses social media Wechat to obtain health information in china: a survey study. BMC Med Inform Decis Mak.

[11] China Internet Network Information Center. The 47th China Statistical Report on Internet Development. 2021. http://cnnic.cn/gywm/xwzx/rdxw/20172017_7084/202102/t20210203_71364.htm/. Accessed 3 Feb 2021.

[12] Liu H, Wang W, Chen H, Li Z, Feng S, Yuan Y (2020). Can WeChat group-based intervention reduce reperfusion time in patients with ST-segment myocardial infarction? a controlled before and after study. J Telemed Telecare.

[13] Zhang X, Xiao H, Chen Y (2019). Evaluation of a WeChat-based life review programme for cancer patients: a quasi-experimental study. J Adv Nurs.

[14] Mao L, Lu J, Zhang Q, Zhao Y, Chen G, Sun M (2019). Family-based intervention for patients with type 2 diabetes via WeChat in China: protocol for a randomized controlled trial. BMC Public Health.

[15] Korda H, Itani Z (2013). Harnessing social media for health promotion and behavior change. Health Promot Pract.

[16] Sun M, Yang L, Chen W, Luo H, Zheng K, Zhang Y (2021). Current status of official WeChat accounts for public health education. J Public Health (Oxf).

[17] Li J, Huang W, Gao J, Li D, Xu L, Huang J (2019). Impact of mobile-based health education on the awareness and knowledge of glaucoma in Chinese patients. Telemed J.

[18] Stellefson M, Paige SR, Chaney BH, Chaney JD (2020). Evolving role of social media in health promotion: updated responsibilities for health education specialists. Int J Environ Health Res.

[19] WHO. Coronavirus disease (COVID-2019) situation report-161. Geneva, World Health Organization, 2020. https://www.who.int/emergencies/diseases/novelcoronavirus2019/situation-reports. Accessed 3 June 2020.

[20] De Laval F, Maugey N, Bonet d'Oléon A, Pommier de Santi V, Ficko C (2021). Increased risk of severe malaria in travellers during the COVID-19 pandemic. J Travel Med.

[21] Xiao S, Li T, Zhou W, Shen M, Yu Y (2020). WeChat-based mHealth intention and preferences among people living with schizophrenia. PeerJ.

[22] Gao XT, Huang KC, Cui X, Zhou CM (2021). WeChat-assisted health education improves care ability, reduces care burden and improves quality of life of parents of infants after enterostomy. J Pediatr Child Health Care.

[23] Bian D, Shi Y, Tang W, Li D, Han K, Shi C (2021). The influencing factors of nutrition and diet health knowledge dissemination using the WeChat official account in health promotion. Front Public Health.

