# Supplementary material for: Using social media for health education and promotion: a pilot of WeChat-based prize quizzes on China national malaria day
Source: Malar J. 2022 Dec 13;21:381. doi: 10.1186/s12936-022-04404-2 (PMC9745723; doi:10.1186/s12936-022-04404-2)
Supplement: Supplementary file 1 — Additional file 1. Question Bank. [file 12936_2022_4404_MOESM1_ESM.docx]

Question Bank

1. What is malaria also called? (A)

A. Da Bai Zi B. Cold C. Fever D. Diarrhea

2. Malaria is a disease caused by infection with what? (C)

A. Bacteria B. Viruses C. Parasites D. Chlamydia

3. How is malaria commonly spread? (A)

A. Mosquito bites B. Inhalation of droplets

C. Eating food contaminated with flies D. Sexual contact

4. In which year does China plan to eliminate malaria? (A)

A. 2020 B. 2025 C. 2030 D. 2035

5. What kind of mosquito spreads malaria? (C)

A. Aedes mosquito B. Culex C. Anopheles

6. Which are malaria endemic areas? (D)

A. Europe B. North America C. Japan D. Southeast Asia and Africa

7. Which of the following symptoms are most likely to occur with malaria infection? (A)

A. Chills-fever, sweating-fever B. Abdominal pain and diarrhea

C. Itchy rash D. Dizziness and headache

8. What action should you take if you are infected with malaria? (B)

A. It's not a big problem, it's just a cold. I can manage it at home by myself.

B. It may be serious if I don’t seek medical treatment in time when infected with malaria. I should go to hospital as soon as possible.

C. Malaria is a chronic disease. I should deal with other important things first. It doesn’t matter if I delay medical treatment.

9. What should I do if I have a fever after returning from Africa or Southeast Asia? (B)

A. I just have a cold, it's not serious.

B. It may be malaria, so I need to go to hospital for a check-up. I should tell the doctor that I travelled to Africa or Southeast Asia.

C. It may be malaria. I need to go to the hospital for a check-up. I can hide my travel information because it is private.

10. Can blood transfusions cause malaria? (B)

A. The blood products we import are strictly tested and are unlikely to be infected with malaria.

B. Although the blood products we transfuse are strictly tested, there is still the possibility of acquiring malaria.

11. Which is the correct option for donating blood? (C)

A. I have been to malaria-endemic areas such as Africa and Southeast Asia in the past year.

B. I have had malaria before, but it has been cured for 1 year.

C. I have had malaria before, but has been cured for 3 years.

D. Although I have had malaria, I can donate blood as long as I am cured.

12. Which option is correct for malaria prevention? (D)

A. Get vaccinated before going to malaria-endemic areas

B. Use mosquito nets in malaria endemic areas

C. Use repellents to prevent mosquito bites

D. All of the above

13. Which day is National Malaria Day? (C)

A. December 1 B. April 25 C. April 26 D. May 1

14. What is the topic for National Malaria Day this year? (B)

A. Eliminate malaria and beware of importation

B. Eliminate malaria and beware of imported re-transmission

C. Eliminate malaria, share health

D. Action for all to eliminate malaria

15. Which type of malaria is the deadliest? (A)

A. *Plasmodium falciparum* B. *Plasmodium vivax*

C. *Plasmodium malariae* D. *Plasmodium ovale*

16. What harm can malaria cause to pregnant women? (B)

A. Dystocia B. Miscarriage, preterm birth or stillbirth

C. Hypertension D. Oedema

17. Which answer is correct about Africa? (D)

A. There is poor sanitation, many mosquitoes and a high incidence of malaria in many parts of Africa.

B. One child dies of malaria every 2 minutes in Africa.

C. Although I only stayed in Africa for 1 night, it is possible to get malaria.

D. All of the above

18. When did China report no locally acquired malaria cases for an entire year ? (C)

A. 2015 B. 2016 C. 2017 D. 2018

19. Which one is correct? (B)

A. There are currently no cases of malaria in China.

B. At present, malaria cases are mainly imported into China.

C. Imported malaria cases are unlikely to cause domestic transmission.

D. Imported malaria cases are few and do not require much investment.

20. What are the isolation treatment requirements for malaria patients? (B)

A. Not required

B. Need to pay attention to anti-mosquito measures

C. Wear a mask to prevent respiratory transmission

D. Need to wear personal protective clothing to prevent contact transmission

21. Which of the following statements about malaria treatment is false? (C)

A. With timely treatment it can be cured

B. Standardized treatment is necessary

C. There are often sequelae after treatment

D. Current medicines for malaria are highly effective

22. What precautions should be taken when treating people infected with malaria? (B)

A. Check-up with family members

B. Fellow travelers should also be tested for malaria when returning

C. People from the same village also need check-up

D. None

23. What tests are needed for diagnosing malaria? (B)

A. Stool test B. Blood test

C. Urine test D. Sputum test

24. When was Jiangsu Province formally assessed as having eliminated malaria?(D)

A. 2016 B. 2017 C. 2018 D. 2019
