# Supplementary material for: Using social media for health education and promotion: a pilot of WeChat-based prize quizzes on China national malaria day
Source: Malar J. 2022 Dec 13;21:381. doi: 10.1186/s12936-022-04404-2 (PMC9745723; doi:10.1186/s12936-022-04404-2)
Supplement: Supplementary file 2 — Additional file 2. Malaria knowledge points. [file 12936_2022_4404_MOESM2_ESM.docx]

Malaria knowledge points

1. What is malaria?

Malaria is a parasitic disease that seriously endangers human health and is commonly known as “Da Bai Zi.” There are four main species of Plasmodium that can infect humans: *Plasmodium falciparum*, *Plasmodium vivax*, *Plasmodium ovale* and *Plasmodium malariae*, of which *Plasmodium falciparum* is the deadliest. The typical symptoms of malaria are a chills-fever, sweating-fever pattern with intermittent retreat of symptoms. Malaria is a statutory Class B infectious disease in China.

1. How does malaria spread?

The main route of transmission of malaria is through the bite of *Anopheles* mosquitoes. Blood transfusion also carries the risk of infection with malaria. To reduce the risk, the "Health Check Requirements for Blood Donors" in China stipulates that individuals who have travelled to malaria-endemic areas within 1 year or who have recovered from malaria within 3 years are temporarily unable to donate blood.

1. How does malaria harm human health?

Malaria is parasitic in human red blood cells, and can cause anemia and splenomegaly. *Plasmodium falciparum* can cause severe malaria and death. Malaria infection in pregnant women can lead to miscarriage, premature birth and stillbirth. It is necessary to seek medical treatment in time when infected with malaria. At present, most of the malaria deaths in China are caused by delayed medical treatment.

1. What is the treatment and prognosis of malaria?

The main drugs for the treatment of malaria include artemisinin, chloroquine, and primaquine. Their curative effect is good and malaria can be cured if treat timeously. You should seek medical treatment in time to prevent severe malaria if you have a fever and have recently returned from Africa or Southeast Asia. Doctors can more effectively make a diagnosis if they are informed by you about travelling abroad. Fellow travelers should also be tested for malaria on return.

1. Where is malaria endemic?

Malaria imposes a severe economic burden on health and one child dies of malaria every 2 minutes in Africa. Malaria is currently widespread in Africa and Southeast Asia. Malaria previously had a serious impact on the lives of individuals in China. China has achieved a status of no local infection cases reported throughout the year since 2017 and planned to achieve the goal of eliminating malaria in 2020. Jiangsu Province was certified as malaria free in 2019. All malaria cases in China are now imported. There is some risk of re-transmission of malaria.

1. How is malaria prevented?

Preventing mosquito bites can effectively prevent malaria. There is no effective vaccine against malaria presently. Malaria can be effectively prevented by taking preventive medicines in advance, and by using mosquito nets and mosquito repellents in endemic areas.

1. What is National Malaria Day?

April 26 is National Malaria Day. The topic is "Eliminate Malaria, Beware of Imported Re-transmission From Overseas" this year.
